# Student Engagement from the Medical Trainees’ Perspective and Associated Factors: A Nationwide Cross-Sectional Study

**DOI:** 10.31662/jmaj.2025-0448

**Published:** 2026-02-20

**Authors:** Hirohisa Fujikawa, Hidetaka Tamune, Yuji Nishizaki, Hirotake Mori, Sho Fukui, Kiyoshi Shikino, Taro Shimizu, Yu Yamamoto, Hiroyuki Kobayashi, Toshio Naito, Yasuharu Tokuda

**Affiliations:** 1 Department of General Medicine, Juntendo University Faculty of Medicine, Bunkyo-ku, Tokyo, Japan; 2 Department of Medical Education Studies, International Research Center for Medical Education, Graduate School of Medicine, The University of Tokyo, Bunkyo-ku, Tokyo, Japan; 3 Center for General Medicine Education, School of Medicine, Keio University, Shinjuku-ku, Tokyo, Japan; 4 Department of Psychiatry and Behavioral Science, Juntendo University Graduate School of Medicine, Bunkyo-ku, Tokyo, Japan; 5 Division of Medical Education, Juntendo University School of Medicine, Bunkyo-ku, Tokyo, Japan; 6 Department of Emergency and General Medicine, Kyorin University, Mitaka, Tokyo, Japan; 7 Division of Rheumatology, Inflammation, and Immunity, Brigham and Women’s Hospital and Harvard Medical School, Boston, MA, USA; 8 Department of Community-Oriented Medical Education, Graduate School of Medicine, Chiba University, Chiba, Chiba, Japan; 9 Department of Diagnostic and Generalist Medicine, Dokkyo Medical University Hospital, Shimotsuga-gun, Tochigi, Japan; 10 Division of General Medicine, Center for Community Medicine, Jichi Medical University, Shimotsuke, Tochigi, Japan; 11 Department of Internal Medicine, Mito Kyodo General Hospital, University of Tsukuba, Mito, Ibaraki, Japan; 12 Muribushi Okinawa for Teaching Hospitals, Urasoe, Okinawa, Japan; 13 Tokyo Foundation for Policy Research, Minato-ku, Tokyo, Japan

**Keywords:** student engagement, student involvement, undergraduate medical education, medical curriculum, medical trainee

## Abstract

**Introduction:**

Despite mounting recognition of the importance of student engagement in curriculum development, the current status of student engagement from medical trainees’ perspectives has yet to be elucidated. Particularly in Japan, where the educational system places strong emphasis on teacher authority, it is possible that medical student engagement is not promoted as effectively as it could be, and that engagement is not fully perceived by medical students. Thus, we aimed to elucidate the current status of student engagement in curriculum development from the perspective of medical trainees, and to explore factors associated with medical trainees’ perceptions of student engagement.

**Methods:**

We performed a nationwide cross-sectional study in Japan from April to May 2025. Participants were newly entered medical residents who took the General Medicine In-Training Examination postgraduate “Year-0.” They completed an anonymous online self-administered questionnaire. We analyzed the closed-ended questions using descriptive statistics and linear mixed-effects models, and applied inductive content analysis to the open-ended questions.

**Results:**

Of 748 examinees, 428 (57.2%) were included in the analysis. A total of 105 (24.5%) did not perceive that there were student engagement initiatives at their medical school. Only 38 (8.9%) reported participation. The trainees’ overall perceptions of how well their opinions were reflected in the medical curriculum were moderate, with a mean score of 5.41 out of 10. This result was supported by the content analysis findings, which identified four themes, including “desire to see more of medical students’ opinions reflected” and “disappointment that medical students’ opinions are not reflected.” Multilevel analysis demonstrated that females had significantly more favorable perceptions than males.

**Conclusions:**

Our findings indicated that awareness, implementation, and perceived value of student engagement remain limited from the perspectives of medical trainees in Japan. Medical educators should implement structural and cultural reforms and develop effective strategies tailored to diverse institutional environments.

## Introduction

Student engagement is defined as “the student investment of time and energy in academic and non-academic experiences that include learning, teaching, research, governance, and community activities” ^(1)^. It encompasses a spectrum of student-educator partnerships aimed at improving educational outcomes, learning environments, and culture within the school. Conceptually, it is often divided into two categories: engagement in learning and engagement in school. Engagement in school extends beyond classroom initiatives to more substantive roles, including participation in policy-making processes, provision of education, and involvement in school governance. In these contexts, student engagement in medical curricula has recently gained increasing momentum in global conversations in the field of medical education. It is likely associated with greater well-being, decreased burnout, and enhanced self-directed learning ^(2), (3), (4)^. In addition, it likely promotes teacher satisfaction and well-being ^(5)^. Consequently, it has been adopted as an indicator of the quality of medical programs and a measure of institutional excellence in medical education ^(6), (7)^. Numerous international and national associations, including the World Federation for Medical Education (WFME), underscore the importance of student engagement ^(7), (8)^.

Despite the growing recognition of the importance of student engagement in medical curricula, empirical studies investigating the current status of engagement from medical trainees’ perspectives and their perceptions of student engagement remain limited ^(9), (10)^. Since medical trainees are important stakeholders, shedding light on their voices is critical. From the perspectives of faculty members, the available reports have primarily focused on student engagement in the context of governance and curriculum reform, frequently underscoring the value of involving students as partners in decision-making and quality-assurance processes. For example, Geraghty et al. ^(10)^ described how medical schools are beginning to empower students as agents of curricular change while also noting that systematic frameworks for such involvement remain underdeveloped. However, faculty perspectives do not necessarily capture how engagement is actually experienced by trainees; international studies in the context of medical education demonstrate that faculty and learners frequently hold divergent views ^(11), (12)^. This highlights the importance of learner-reported data, which are not interchangeable with faculty surveys and are essential to understanding engagement as a learner-centered construct. To date, however, national-level studies of student voices are rare ^(13)^. This gap is critical because student perceptions likely have a considerable impact on engagement behaviors; if students perceive engagement as burdensome and irrelevant, medical school efforts to promote student engagement may fail. Accordingly, elucidating medical trainees’ voices on engagement is a high-priority subject.

In Japan, the importance of student engagement in medical curricula has attracted considerable attention. Indeed, its value is highlighted by the Japan Accreditation Council for Medical Education (JACME), the organization recognized by the WFME to assess medical education programs at Japanese universities ^(14)^. JACME adheres to Japanese standards that are based on the WFME Basic Medical Education standards ^(8), (15)^. The Japanese standards encompass program evaluation and governance and call for policies that involve students in key aspects of school management and quality assurance, thereby embedding student engagement within the national quality-assurance agenda ^(8)^. However, given that the Japanese educational system and teacher-student relationship are characterized by strong teacher authority ^(16)^, it is possible that medical student engagement in Japan is not promoted to the degree expected, and that engagement is not fully perceived by medical students.

Accordingly, our research questions were as follows: (1) What is the current status of student engagement from the perspectives of medical trainees?; and (2) what factors are associated with medical trainees’ perceptions of student engagement? Unlike many previous reports that focused on institutional or faculty perspectives, this study is the first, to our knowledge, to specifically target recent trainees to capture their firsthand perceptions regarding student engagement, and therefore addresses a critical gap in the literature. The present study provides a trainee-reported national baseline on engagement in medical curriculum development, furnishing benchmarks that can guide future comparisons across stakeholder groups and inform strategies to strengthen student engagement.

## Materials and Methods

### Design, setting, and participants

We conducted this nationwide cross-sectional study as a component of a series of research projects on student engagement and extracurricular activities in Japanese undergraduate medical education. We distributed an online anonymous self-administered questionnaire to medical trainees who participated in the General Medicine In-Training Examination postgraduate “Year-0” (GM-ITE PGY-0) from April 4 to May 31, 2025. In the Japanese medical education system, medical trainees usually graduate from university in March and enter their residency program in April ^(15)^. Consequently, the timing of our survey was just after graduation from medical school, making it appropriate to ask about the content of undergraduate medical education ^(17)^. The GM-ITE PGY-0 was developed in 2018 by the Japan Institute for Advancement of Medical Education Program (a non-profit organization). It is administered just after the initiation of residency training to capture data on trainees at the beginning of their residency and is now taken by many medical trainees across Japan ^(17)^.

Following completion of the GM-ITE PGY-0, the examinees were asked to participate in the study. Prior to participation, they read a document that described the anonymity and voluntary nature of the study. Only participants who provided informed consent were included in the analysis. Because we intended to survey the current undergraduate medical education system in Japan, we excluded trainees from foreign medical schools.

### Questionnaire

We developed a questionnaire that asked about medical trainees’ perceptions of student engagement with reference to previous literature (File S1) ^(1), (10), (18)^. The first three questions inquired about the participant’s prior experiences regarding student engagement using closed-ended questions. To elicit additional information, we also offered an optional open-ended question regarding student engagement or related factors.

### Data analysis

In this study, we analyzed the data quantitatively and qualitatively as follows.

To quantify baseline trainee-reported prevalence of awareness and participation in student engagement in medical curricula, we first computed descriptive statistics for closed-ended questions as national reference values. Additionally, to test the primary hypothesis that perceptions of how well medical students’ opinions were reflected in the medical curriculum differ by individual or environmental factors, we employed a linear mixed-effects model (random intercept model) that included a random effect for alma mater medical school and covariates (sex, age, university location, and university type) as fixed effects. Because the primary analysis suggested that females demonstrated more positive perceptions than males (as will hereinafter be described in detail), we then conducted exploratory subgroup analyses to assess whether the same directional pattern (female > male) was observed within strata of age, university type, and university location; given the exploratory nature, no multiplicity adjustments were applied. All quantitative analyses were performed using SPSS version 29.0.2.0 (IBM Corp). All tests were two-tailed, with p < 0.05 considered significant.

We analyzed free-text responses utilizing inductive content analysis using Haggarty et al.’s ^(19)^ definition of content analysis, namely “a research method which allows the qualitative data collected in research to be analyzed systematically and reliably so that generalizations can be made from them in relation to the categories of interest to the researcher”. We performed content analysis inductively with reference to previous studies ^(20), (21), (22), (23), (24)^: first, the first author read each of the free-text responses multiple times to familiarize himself with the data. Second, he inductively generated initial codes. Third, all authors reviewed the codes. Discussions were held until a consensus was reached. The codes were classified into themes based on similarities. Fourth, the frequency and percentage of each theme were calculated to demonstrate its relative importance in the overall picture. We calculated the percentage using the total number of codes as the denominator. Illustrative quotations were also presented.

### Ethical considerations

We obtained ethical approval from the ethics committee of Japan Institute for Advancement of Medical Education Program (JAMEP) (no. 24-22). All participants provided written informed consent by ticking the consent box in the web questionnaire.

## Results

Of the 748 GM-ITE PGY-0 takers (270 females (36.1%) and 478 males (63.9%)), 471 consented to participate in the study. A total of 12 individuals from medical schools outside Japan were excluded. Of the remaining 459 individuals, 31 were excluded due to missing data, and 428 (response rate: 57.2%) were included in the final analysis. These participants were from 75 of the total 82 medical schools in Japan. We summarize participant profiles in Table 1. Of the 428 participants, 138 (32.2%) were female, and 290 (67.8%) were male. To examine potential non-response bias, we compared the participants who were included and those who were excluded by the only available characteristic, sex, and presented the results in File S2. Sex was the only characteristic available for excluded participants; included participants had a higher proportion of males than excluded participants.

**Table 1.** Characteristics of the Study Participants (N = 428).

Characteristic	Value
Sex, n (%)	
Female	138 (32.2)
Male	290 (67.8)
Age, n (%)	
24	170 (39.7)
25	132 (30.8)
26-30	105 (24.5)
≧31	21 (4.9)
University type, n (%)	
National/public	278 (65.0)
Private	150 (35.0)
University location, n (%)	
Rural	273 (63.8)
Urban	155 (36.2)

Table 2 shows participants’ prior experiences regarding student engagement. A total of 105 (24.5%) participants were not aware that there were student engagement initiatives at their alma mater medical school. Of the 38 participants who participated in student engagement initiatives, 16 (42.1%) were involved on a voluntary basis. In the global rating scale that inquired about the perception of the extent to which medical students’ opinions were reflected in the medical curriculum (from 0 = worst possible to 10 = best possible), the most frequent choice was 5 (75 out of 428 participants, 17.5%), with a mean of 5.41 and standard deviation (SD) of 2.47.

**Table 2.** Participant Responses to Closed-Ended Style Questions That Asked About Their Prior Experiences in Student Engagement (N = 428).

Question	n (%)
“Please indicate whether or not you were involved in student engagement at your alma mater medical school.”	
There were no such initiatives at my alma mater medical school.	105/428 (24.5%)
There were such initiatives at my alma mater medical school, but I did not participate.	285/428 (66.6%)
There were such initiatives at my alma mater medical school, and I participated.	38/428 (8.9%)
“If you chose ‘There were such initiatives at my alma mater medical school, and I participated.’ in the previous question, why did you get involved in the initiatives?”	
Involved voluntarily	16/38 (42.1%)
Elected in an election	1/38 (2.6%)
Elected by random lottery	9/38 (23.7%)
Other	
Invited to participate by faculty members	3/38 (7.9%)
Participation triggered by questionnaire responses	3/38 (7.9%)
Concurrent position with another public committee	1/38 (2.6%)
Mandatory participation	1/38 (2.6%)
Unclear	4/38 (10.5%)
“Using any number from 0 to 10, where 0 is the worst possible, and 10 is the best possible, what number would you use to rate the extent to which medical students’ opinions were reflected in the medical curriculum at your alma mater medical school?” (mean, 5.41; standard deviation, 2.47)	
0	18/428 (4.2%)
1	18/428 (4.2%)
2	18/428 (4.2%)
3	48/428 (11.2%)
4	35/428 (8.2%)
5	75/428 (17.5%)
6	51/428 (11.9%)
7	73/428 (17.1%)
8	57/428 (13.3%)
9	21/428 (4.9%)
10	14/428 (3.3%)

Table 3 shows the results of multilevel analysis after adjustment for clustering within medical schools to explore whether individual or environmental factors were associated with the perception of the extent to which medical students’ opinions are reflected in the curriculum. Females demonstrated more positive perceptions than males (adjusted mean difference: 0.69, 95% confidence interval (CI): 0.23-1.12). Other factors were not associated with the perception of the extent to which medical students’ opinions are reflected in the curriculum. File S3 shows the results of the exploratory subgroup analyses. Across all strata, the point estimates for females were all positive (i.e., female trainees tended to report more positive perceptions than their male counterparts), indicating directional consistency. Statistical significance―defined by 95% CIs excluding zero―was observed among trainees aged 25 years or older, those in private universities, and those in urban universities. In the remaining subgroups, the point estimates remained positive, but the CIs crossed zero.

**Table 3.** Results of a Multilevel Analysis That Explored Whether Individual or Environmental Factors Were Associated with Perceptions on the Extent to Which Medical Students’ Opinions Are Reflected in the Medical Curriculum^a^ (N = 428).

	Adjusted mean difference	95% confidence interval
Sex		
Male	Ref.	Ref.
Female	0.69	0.23 to 1.12^**^
Age		
24	Ref.	Ref.
25	-0.32	-0.84 to 0.20
26-30	-0.11	-0.69 to 0.48
≥31	-0.33	-1.37 to 0.72
Type of university		
National/public	Ref.	Ref.
Private	-0.39	-1.10 to 0.32
Location of the university		
Rural	Ref.	Ref.
Urban	0.31	-0.37 to 1.00

Ref.: reference category. ^a^A random intercept model. ^**^p < 0.01.

Six trainees provided responses to the free-text item regarding student engagement. We generated one code for each trainee. Table 4 shows the result of inductive content analysis. As indicated by the emergence of the themes, “desire to see more of medical students’ opinions reflected,” “disappointment that medical students’ opinions are not reflected,” and “necessity of creating an atmosphere that promotes student engagement,” there were many requests for improvements regarding university initiatives related to student engagement.

**Table 4.** Opinions, thoughts, or suggestions regarding medical student engagement.

Theme	n (%)^a^	Illustrative quotes
Desire to see more of the medical students’ opinions reflected	2 (33.3)	“I hope that the program will incorporate even more student opinions than before.”
Disappointment that medical students’ opinions are not reflected	2 (33.3)	“I don’t feel like my opinions are being considered.”
Necessity of creating an atmosphere that promotes student engagement	1 (16.7)	“It is necessary to create an atmosphere that promotes student engagement.”
Improved learning due to student engagement	1 (16.7)	“I believe that when medical students actively participate in their education, they can better focus on their learning objectives.”

^a^The percentages were calculated using a denominator of 6 (total number of the codes).

## Discussion

This nationwide cross-sectional study is, to our knowledge, the first to investigate the current status of student engagement from the perspective of medical trainees and to explore factors that were associated with their perceptions of student engagement. The study produced several noteworthy findings. First, awareness of and participation in student engagement were limited. Nearly a quarter of the participants did not perceive that there were student engagement initiatives at their alma mater medical school, and less than 10% reported participation. Additionally, among those who did join, less than half did so on a voluntary basis. Second, trainees’ overall perceptions of how well their opinions were reflected in the medical curriculum were moderate, with a mean score of 5.41 out of 10. This finding was supported by the results of the qualitative analysis. Third, the multilevel analysis showed that females reported significantly more favorable perceptions. Thus, these findings indicated that whereas student engagement is internationally recognized as an important determinant of quality medical education, its awareness, implementation, and perceived value remain limited among medical trainees in Japan.

Despite JACME’s emphasis on student engagement in medical curricula, our findings suggest limited uptake: approximately one-quarter of participants were not even aware of engagement initiatives, fewer than one in 10 reported any participation, and less than half of those who participated did so voluntarily. This is noteworthy, as a recent scoping review highlighted that the majority of reports on student engagement in curriculum development are from Europe and North America, with few examples from other regions, particularly in the Global South, where specific cultural dimensions such as collectivism and high-power distance influence student-teacher relationships, learner involvement, and the learning environment ^(25), (26)^. Although Japan is not part of the Global South, its traditionally hierarchical medical education system may similarly concentrate decision-making among faculty and administrators, thereby possibly leaving limited space for student involvement ^(9)^. As Barzansky and Fuentealba have indicated, a successful student engagement program necessitates a supportive organizational culture and learning environment in which students are regarded as partners ^(27)^. Accordingly, Japanese medical schools should implement structural reforms that will foster formal avenues for student engagement and promote cultural shifts that recognize students as partners in medical education, rather than passive recipients ^(28), (29)^.

This multilevel analysis showed significant differences in perceptions regarding the extent to which trainees’ opinions were reflected in the medical curriculum based on sex. Female trainees reported significantly more favorable perceptions regarding the extent to which their opinions were reflected in the curriculum compared to male students (adjusted mean difference: 0.69, 95% CI: 0.23-1.12). In subgroup analyses, this directional pattern was consistent across age, university type, and university location: point estimates were positive in all strata, with 95% CIs excluding zero in urban and private universities and among trainees aged ≥25 years, whereas the corresponding CIs crossed zero in rural, public/national, and age-24 strata. However, given the overall SD of 2.47, the adjusted mean difference corresponds to approximately 0.3 SD, indicating a small effect size. The response distribution was centered at 5 with 7 as the next most frequent value. Accordingly, the magnitude should be interpreted as modest and possibly influenced by response-scale preferences (e.g., midpoint use) rather than meaningful differences in perception.

Nonetheless, even such small differences could reflect subtle structural or cultural dynamics shaping how trainees perceive their influence. In the 2025 Global Gender Gap Index, which places 148 countries on a scale of gender equality, Japan ranked 118th place, the lowest among G7 countries ^(30)^. Within medicine, female physicians and medical trainees have also been underrepresented ^(31), (32)^. Nevertheless, as Watari et al. ^(33)^ indicated, a significant improvement has been observed in gender equality in medical school admissions, particularly following a 2018 scandal involving the manipulation of scores in several Japanese medical schools ^(34), (35)^. This improvement may be attributable to the endeavors of medical faculty members to address issues of gender inequality. A recent scoping review highlighted that stakeholder open-mindedness toward students is a key determinant of meaningful student engagement, whereas a lack of openness and entrenched power distance can suppress student voice ^(25)^. Although our data do not allow causal interpretation, this societal context may help explain why female trainees, among respondents, reported somewhat more favorable perceptions of how their opinions were reflected in the curriculum. Such perceptions could reflect growing awareness or receptivity within faculty-student relationships. Importantly, the female-male difference was evident across diverse settings, although it achieved statistical significance only in specific strata (urban, private, and older trainees). This pattern may reflect limited sample size and statistical power in certain subgroups. The findings suggest that faculty and schools should continue to develop structures and practices that make engagement opportunities equitably visible and accessible across institutional and demographic contexts.

Our qualitative analysis of free-text responses revealed trainees’ nuanced perspectives on student engagement, which in turn emphasized and strengthened the quantitative findings. Due to the limited qualitative sample, these themes may reflect the views of a highly selective subset and should likely not be overgeneralized. Nevertheless, the emergent themes indicated a sense of frustration among medical trainees regarding their limited impact within medical education, alongside a strong desire for more meaningful opportunities to be involved in initiatives that shape their learning environment. This is consistent with a previous qualitative study in Japan targeted at medical students who worked to improve educational processes at their medical school. That study demonstrated that students were disappointed that their efforts had not resulted in improvements to the medical curriculum and their perceived obligation to serve the public ^(9)^. The findings suggested that the voices of medical students, despite their status as a pivotal stakeholder group, may not be adequately addressed. We medical educators should cultivate a supportive organizational culture and learning environment in which medical students are treated as partners. Such a culture and environment would benefit both learners themselves (e.g., greater self-confidence) and their school (e.g., learners’ greater commitment to institutional success) ^(27), (36), (37)^.

This study had several potential limitations. First, there may have been a degree of selection bias. It is possible that GM-ITE PGY-0 takers are highly motivated trainees. If so, we were unable to include trainees with lower learning motivation. Second, only sex was available for excluded participants, preventing comparisons on other attributes. There may be potential non-response bias. Third, the study design has a potential risk of recall bias. Fourth, the global rating scale that assessed perceptions regarding the extent to which trainees’ opinions were reflected in the curriculum was not validated. Additionally, although we reported a mean of 5.41 (SD: 2.47) and a mode of 5, there are no widely accepted cutoffs for interpreting 0-10 global rating scales in medical education. Our description of the score as “moderate” was therefore descriptive rather than based on a validated threshold. Future studies should employ validated instruments or establish the psychometric properties of newly developed scales and should also work toward developing empirically grounded interpretive benchmarks for global rating data to ensure reliable assessment of trainee perceptions. Fifth, a few participants provided a free-text response. As described above, highly motivated trainees may have responded: because the survey was distributed following a two-hour examination, the participants may have suffered from fatigue, resulting in few responses to the open-ended question. Sixth, although we employed a linear mixed-effects model in this cross-sectional study, the study design precludes causal inference. In this regard, future research should employ longitudinal approaches. Despite these limitations, our study had several strengths. First, to our knowledge, this is the first empirical study to examine the current status of student engagement from medical trainees’ perspectives and their perceptions of student engagement. Because medical trainees are a crucial but often overlooked stakeholder, our findings of the voices of these stakeholders are notable. Second, we covered 75 of the total of 82 Japanese medical schools using the JAMEP nationwide network. Third, the response rate of this study was 57.2%, which was greater than the criterion considered desirable for online surveys ^(38)^. The insights provided by this study will inform institutional strategies aimed at fostering meaningful student involvement in medical education.

### Conclusions

In this study, we investigated Japanese medical trainees’ perceptions of student engagement in undergraduate medical education and explored associated factors. The findings indicate that, despite international recognition of the importance of student engagement in medical curricula, its awareness, implementation, and perceived value remain limited from the perspective of medical trainees in Japan. Medical educators should promote structural and cultural reforms in which students are treated as partners in medical education. Further, they should design effective strategies tailored to diverse institutional environments, with consideration to sociohistorical and contextual factors. The present study provides a trainee-reported national baseline, offering benchmarks for cross-stakeholder comparisons and guidance to strengthen student engagement.

## Article Information

### Acknowledgments

The authors express their gratitude to the GM-ITE postgraduate “Year-0” examinees for participating in the study. The authors would also like to thank ChatGPT-5 from OpenAI for its valuable assistance in refining the academic writing.

### Author Contributions

Conceived the study: Hirohisa Fujikawa, Hidetaka Tamune, Yuji Nishizaki, Hirotake Mori, Sho Fukui, Kiyoshi Shikino, Taro Shimizu, Yu Yamamoto, Hiroyuki Kobayashi, Toshio Naito, and Yasuharu Tokuda. Conducted the data analysis: Hirohisa Fujikawa, which was reviewed by Hidetaka Tamune, Yuji Nishizaki, Hirotake Mori, Sho Fukui, Kiyoshi Shikino, Taro Shimizu, Yu Yamamoto, Hiroyuki Kobayashi, Toshio Naito, and Yasuharu Tokuda. Drafted the manuscript: Hirohisa Fujikawa. Discussed, proofread, and approved the final version of the manuscript: all authors.

### Conflicts of Interest

Hidetaka Tamune, Sho Fukui, Kiyoshi Shikino, Taro Shimizu, and Yu Yamamoto have received honoraria from the Japan Institute for Advancement of Medical Education Program (JAMEP) as exam preparers of the General Medicine In-Training Examination (GM-ITE). Yuji Nishizaki has received an honorarium from JAMEP as the GM-ITE project manager. Kiyoshi Shikino and Hiroyuki Kobayashi have received honoraria from JAMEP as speakers at JAMEP lectures. Yasuharu Tokuda is the director of JAMEP and has received an honorarium from JAMEP as a speaker at a JAMEP lecture. Hidetaka Tamune, Yuji Nishizaki, Sho Fukui, Kiyoshi Shikino, Taro Shimizu, Yu Yamamoto, Hiroyuki Kobayashi, and Yasuharu Tokuda were not involved in the data analysis. Otherwise, the authors declare that they have no conflict of interest.

### Ethical Approval Statement

This study was conducted in accordance with the Declaration of Helsinki and relevant guidelines. We obtained ethical approval from the ethics committee of the JAMEP (no. 24-22). All participants provided written informed consent by ticking the consent box in the web questionnaire.

## Supplement

Supplementary Material
