# Supplementary material for: Student Engagement from the Medical Trainees’ Perspective and Associated Factors: A Nationwide Cross-Sectional Study
Source: JMA J. 2026 Feb 20;9(2):486–94. doi: 10.31662/jmaj.2025-0448 (PMC13061581; doi:10.31662/jmaj.2025-0448)
Supplement: Supplementary Material [file 2433-3298-9-2_0486-s001.pdf]

File S1. Content of the questionnaire

In recent years, the importance of student engagement in medical education—initiatives where medical students participate in their own educational system, such as reflecting student opinions in the medical curriculum—has gained attention. In the following questions, please indicate the situation regarding student engagement at your alma mater medical school.

Q1. Please indicate whether or not you were involved in student engagement at your alma mater medical school.

1. There were no such initiatives at my alma mater medical school.
2. There were such initiatives at my alma mater medical school, but I did not participate.
3. There were such initiatives at my alma mater medical school, and I participated.

Q2. If you chose "There were such initiatives at my alma mater medical school, and I participated" in the previous question, why did you get involved in the initiatives?

1. Involved voluntarily
2. Elected in an election
3. Elected by random lottery
4. Other

Q3. Using any number from 0 to 10, where 0 is the worst possible and 10 is the best possible, what number would you use to rate the extent to which medical students' opinions were reflected in the medical curriculum at your alma mater medical school?

1. 0
2. 1
3. 2
4. 3
5. 4
6. 5
7. 6
8. 7
9. 8
10. 9
11. 10

Q4. Please feel free to describe any opinions, thoughts, or suggestions regarding medical student engagement.

File S2. Comparison of the Participants Who Were Included and Those Who Were Excluded, by Sex.

	Included participants, 428 (57.2%)	Excluded participants, 320 (42.8%)	P value[a]
Female, 270 (36.1%)	138	132	0.014
Male, 478 (63.9%)	290	188	

[a] P value by chi-square test

File S3. Subgroup Analysis of the Female–Male Gap in Perceived Curricular Influence.[a]

Subgroup	Adjusted mean difference (female – male)	95% confidence interval
Urban	0.92	0.15 to 1.69*
Rural	0.54	-0.04 to 1.12
Private university	1.22	0.37 to 2.08**
National/public university	0.34	-0.20 to 0.88
Age 24	0.67	-0.01 to 1.34
Age ≥ 25	0.77	0.14 to 1.41*

[a] Random intercept model

* $p < 0.05$, ** $p < 0.01$
